# Sea Urchin as a Universal Model for Studies of Gene Networks

**DOI:** 10.3389/fgene.2020.627259

**Published:** 2021-01-20

**Authors:** Leonid Adonin, Anatoliy Drozdov, Nickolai A. Barlev

**Affiliations:** ^1^Moscow Institute of Physics and Technology, Dolgoprudny, Russia; ^2^Institute of Environmental and Agricultural Biology (X-BIO), Tyumen State University, Tyumen, Russia; ^3^Orekhovich Institute of Biomedical Chemistry, Moscow, Russia; ^4^Zhirmunsky National Scientific Center of Marine Biology, Far Eastern Branch of the Russian Academy of Sciences, Vladivostok, Russia; ^5^Institute of Cytology, Russian Academy of Sciences, Saint-Petersburg, Russia

**Keywords:** sea urchin, gene expression, cell signaling, long non-coding RNA, genomics

## Abstract

The purple sea urchin *Strongylocentrotus purpuratus* has been used for over 150 years as a model organism in developmental biology. Using this model species, scientists have been able to describe, in detail, the mechanisms of cell cycle control and cell adhesion, fertilization, calcium signaling, cell differentiation, and death. Massive parallel sequencing of the sea urchin genome enabled the deciphering of the main components of gene regulatory networks during the activation of embryonic signaling pathways. This knowledge helped to extrapolate aberrations in somatic cells that may lead to diseases, including cancer in humans. Furthermore, since many, if not all, developmental signaling pathways were shown to be controlled by non-coding RNAs (ncRNAs), the sea urchin organism represents an attractive experimental model. In this review, we discuss the main discoveries in the genetics, genomics, and transcriptomics of sea urchins during embryogenesis with the main focus on the role of ncRNAs. This information may be useful for comparative studies between different organisms, and may help identify new regulatory networks controlled by ncRNAs.

## Introduction

The first use of animals for experimental purposes dates back to Ancient Greece. “Generation of Animals” of Aristotle (1942) describes the first systematic study of embryonic development as a phenomenon, which recognizes the key questions about the emergence and relations between hierarchically organized parts of an organism.

Model organisms help in the testing of novel biological hypotheses, which come from *in cellulo* observations and need to be tested at the whole organism level. Hence, model organisms represent a very important tool in modern biology. Currently, the list of model organisms includes over 100 species of animals, plants, protozoa, and viruses. The most popular model species include the frog, zebrafish, сhicken, mouse, fruit fly, and nematode (Rzepnikowska et al., 2017; Kuo et al., 2018; Chatterjee and Deng, 2019; Marques et al., 2019; Nielsen, 2019; Tang et al., 2019). All these species are used by researchers in a wide range of molecular biological applications, but, unfortunately, none of them are versatile enough to satisfy various experimental needs.

In this review, we focus on the main discoveries in genetics and genomics that were made using a popular model object – the purple sea urchin *Strongylocentrotus purpuratus*, which has been used in biology for over 150 years (Stimpson, 1857). The species was chosen as a model object for several objective reasons: sea urchins are easy to propagate in the laboratory; it is easy to get synchronous embryo cultures and induce rapid embryogenesis; the embryo is transparent and has a simple structure. Genome sequencing and the description of complex gene regulatory networks during the sea urchin embryogenesis made this model object indispensable for the study of gene expression regulation.

**Figure 1:**
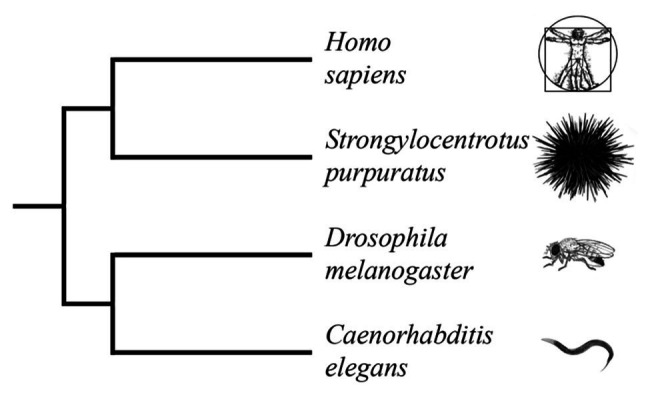
Truncated phylogenetic tree of popular model organisms based on combined analyses of morphology and molecular data (Laumer et al., 2015; Telford et al., 2015; Torruella et al., 2015; Cannon et al., 2016). The tree illustrates the evolutionary relationship between *Homo sapiens* and *Strongylocentrotus purpuratus* as members of the deuterostome branch of the animal kingdom. *Caenorhabditis elegans* and *Drosophila melanogaster* are members of the protostome branch. (Branch lengths are not proportional to time).

Echinoderms are a sister group of the Chordate phylum. This group has branched out from Chordate before the Cambrian period (more than 500 million years ago, Figure 1; McClay, 2011). Other studies, based on the multigene and multiprotein studies, indicate a more accurate time of divergence (Hedges, 2002; Zaidel-Bar, 2009).

**Figure 2:**
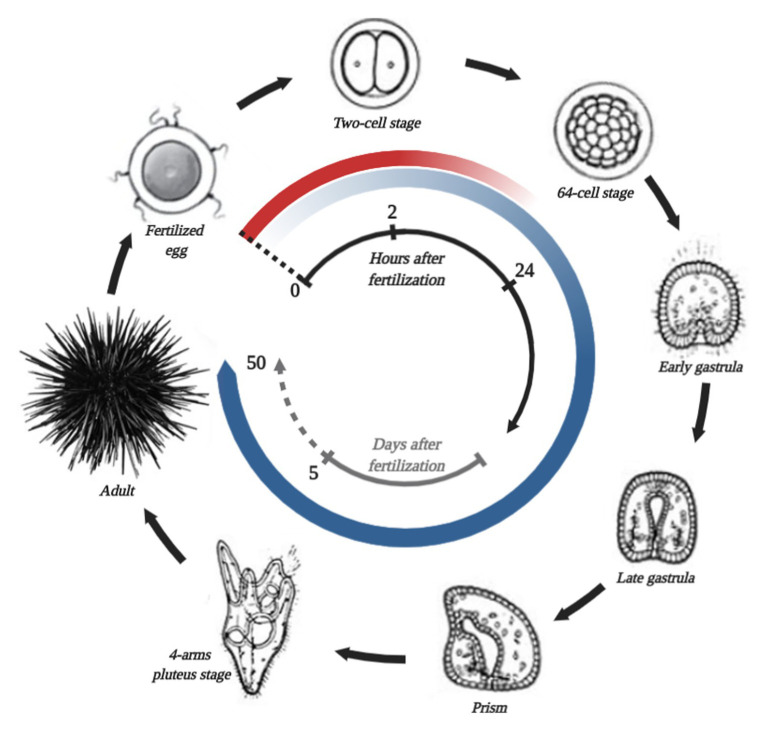
Simplified *S. purpuratus* life cycle, stages of which are connected by black arrows. The name of each stage is shown in the picture. The beginning of the life cycle is fertilization, which is marked by a black dotted line. In the center, the black and gray time scale represents hours and days after fertilization, correspondingly. The red circular gradient line represents the degradation of the general maternal transcripts. The circular blue gradient shows the beginning of zygotic genome activation and increases in transcribed gene numbers.

The first studies, which describe the normal embryogenesis of a sea urchin, date back to the middle of the 19th century. Since then, sea urchin embryos have become a popular model in developmental biology. The normal life cycle of sea urchins is shown in Figure 2. At present, certain echinoid species (e.g., *S. purpuratus*, *Strongylocentrotus droebachiensis*, *Strongylocentrotus intermedius*, *Hemicentrotus pulcherrimus*, *Lytechinus variegatus*, *Paracentrotus lividus*, and *Mesocentrotus franciscanus*) are widely used as experimental models in developmental biology (McClay, 2011). The early stage of embryogenesis of the purple sea urchin was used for studying intercellular communication and cell adhesion (Horstadius, 1939; Giuduce, 1962; McClay and Fink, 1982), cell cycle control mechanisms (Evans et al., 1983), calcium signaling (Whitaker, 2006), fertilization (Briggs and Wessel, 2006), cell differentiation (Giudice, 1973), and cell survival and death (Chiarelli et al., 2016).

The genome size of the purple sea urchin is only a quarter of the human genome, despite it having about the same number of genes. Genome analysis has shown that most of its genes are common to representatives of deuterostomes, which has in turn uncovered an unexpectedly close relation to humans among all used invertebrate model species (Davidson, 2006; Sea Urchin Genome Sequencing Consortium et al., 2006; Cameron, 2014). For example, the sea urchin genome contains orthologs of human disease-associated genes, which are expressed in sea urchin embryogenesis.

The sea urchin genome was shown to contain more than 400 genes, whose products are involved in the regulation of cell homeostasis. Most of these genes display a remarkable conservation of their sequences during the evolution (Goldstone et al., 2006; Rast et al., 2006). The sea urchin genome contains 65 genes of the ATP-binding cassette transporter superfamily (Hamdoun et al., 2004; Goldstone et al., 2006), while, in humans, only 48 members of this family are known to date (Dean and Annilo, 2005). Mutations in these genes lead to several pathologies in humans, including degeneration of the retina, cystic fibrosis, neurological diseases, cholesterol transport disorders, anemia, and many others (Dean et al., 2001). The sea urchin as an experimental model is also frequently used in toxicology and in environmental human health science since it allows an accurate estimate of cancer risk before any epidemiologic evidence is available (Bellé et al., 2007).

Furthermore, sea urchin embryos are used by scientists as a convenient object for elucidating common cellular molecular mechanisms involved in human health and disease. In particular, the sea urchin is used as a model system for studying neurodegenerative disorders that can cause dementia and memory loss (Nakajima et al., 2004).

In the past, it was hypothesized that certain signaling pathways involved in the embryo’s morphogenesis could be aberrantly activated during tumorigenesis. Unfortunately, to develop this idea further, scientists did not have an appropriate human experimental model. This is due to various ethical aspects that restricted human embryo studies (De Wert et al., 2002; De Wert and Mummery, 2003; Holm, 2003; Lo and Parham, 2009). Therefore, an early embryogenesis of sea urchins could be a good model for cancer research.

The normal processes of cell proliferation and differentiation are controlled by several developmental gene regulatory networks. Disunity in these networks leads to the initiation and progression of tumors (Hanahan and Weinberg, 2000; Reya et al., 2001; Pires-daSilva and Sommer, 2003).

**Figure 3:**
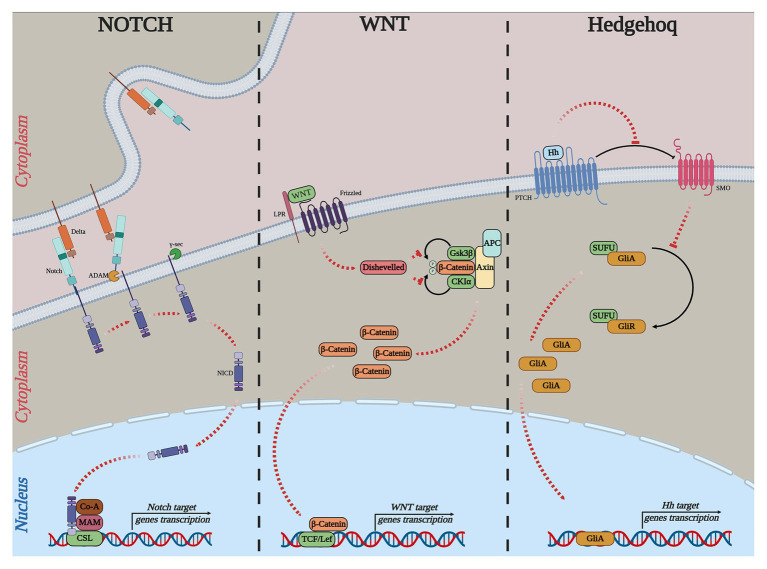
Simple outline of the current models of the canonical Notch, WNT, and Hh pathways. NOTCH: Delta, delta-like ligand, Notch, ADAM, ADAM-family metalloprotease; *γ*-sec, γ-secretase; NICD, notch intracellular domain; Co-A, transcription coactivator; MAM, conserved and essential nuclear factor mastermind; CSL, DNA-binding transcription factor. WNT: Frizzled; WNT, wingless-type MMTV integration site; LRP, low-density lipoprotein receptor-related protein; APC, adenomatous polyposis coli; Disheveled, cytoplasmic phosphoprotein; GSK3ß, glycogen synthase kinase-3; CK1*α*, casein kinase 1 alpha; TCF, T-cell-specific transcription factor; LEF, lymphoid enhancer-binding factor. Hedgehog: Hh, hedgehog; PTCH, patched; SMO, smoothened; SUFU, suppressor of fused; GLI, GLI-family zinc finger.

Notch, Wnt, and Hedgehog (Hh) signaling pathways are highly conserved from sea urchins to humans. The current model of these pathways, including general components shown on Figure 3 and their main roles in embryogenesis and cancer, is described on Table 1.

**Table 1:** Major Notch, WNT, and Hedgehog (Hh) pathways roles in multicellular organisms’ embryo development and cancer.

	NOTCH	WNT	Hedgehog
Functions in development	The Notch pathway is a major determinant of cell fate across all metazoans (Artavanis-Tsakonas and Muskavitch, 2010; Bray, 2016; Henrique and Schweisguth, 2019; Lloyd-Lewis et al., 2019).	The Wnt signaling pathway regulates many cell functions, including proliferation, migration, apoptosis, and differentiation. It also plays a key role in controlling body axis formation. It is essential during embryonic development and also in the homeostasis of several adult tissues including the GI tract (Flanagan et al., 2015, 2017), liver, breast, and skin (Nusse and Clevers, 2017).	The Hedgehog signaling pathway plays a significant role in the normal embryonic development of invertebrates and vertebrates (Skoda et al., 2018). The Hh genes are played in organization of the polarity of the organism and the development of many tissues and organs. The pathway is involved in the maintenance of somatic stem cells and pluripotent cells important for tissue repair (Beachy et al., 2004; Karhadkar et al., 2004; Zhou et al., 2006; Stecca et al., 2007; Lowry et al., 2008).
Role in cancer	Notch plays an oncogenic role: it is overexpressed in breast cancer (Kontomanolis et al., 2018), gastric cancer (Zhou et al., 2013), pancreatic cancer (Ma et al., 2013), and colorectal cancer (Vinson et al., 2016). Notch acts as a tumor suppressor gene: its expression is downregulated in skin cancer (Lefort et al., 2007), liver cancer (Viatour et al., 2011), non-small cell lung cancer (Konishi et al., 2010), and some breast cancers (Parr et al., 2004).	Mutations of Wnt pathway members cause cancer development in humans (Segditsas and Tomlinson, 2006). It is known that Wnt signaling is deregulated in gastric tumors (Clements et al., 2002; Flanagan et al., 2017). The WNT pathway plays critical roles in epithelial ovarian cancer development (Nguyen et al., 2019), colorectal cancer (Wang et al., 2018), and thyroid carcinogenesis (Ely et al., 2018).	Hh signaling is involved in the development of pancreatic, and esophageal cancer (Bailey et al., 2009), gastric, and prostate cancer (Sheng et al., 2004), as well as basal cell carcinoma (Gutzmer and Solomon, 2019) and medulloblastoma (Gordon et al., 2018).

The Hh pathway plays a crucial role in many fundamental processes of metazoan organisms, including tissue homeostasis and their embryonic development. In the development of the sea urchin, this pathway controls the establishment of the left–right asymmetry in embryos (Warner et al., 2016). According to the previously proposed model, this signaling pathway, similar to vertebrates, provides an asymmetrical expression of Nodal, which is an important cytokine of the TGF beta superfamily. Moreover, in humans, the aberrant activation of this signaling pathway is increasingly associated with various cancers. For example, Hh was shown to control proliferation, malignancy, and metastasis (Sari et al., 2018). In particular, while the Hh signaling pathway is mainly repressed during mammary embryonic development, overexpression of some components (PTCH1, GLI1/2) of the Hh are up-regulated in tumor stem cells of human breast cancer (Liu et al., 2006).

The key components of Notch signaling are present in all metazoan organisms (Gordon et al., 2008). The canonical Notch pathway begins when a ligand of the Delta/Serrate/LAG-2 (DSL) family binds to the transmembrane receptor protein, Notch (Fehon et al., 1990). The schema of all stages of the process is shown in Figure 3 (part NOTCH). It is well established that the Delta/Notch signaling pathway is intimately involved in mesoderm formation. Subsequently, deregulation of this pathway leads to the elimination of mesoderm derivatives during the embryogenesis of sea urchins (Sherwood and McClay, 1999, 2001; Sweet et al., 2002; Croce and McClay, 2010). In humans, members of the Notch signaling pathway play a key role in embryonic vasculature development (Patel et al., 2005). Notch can in fact be either oncogenic or tumor suppressive depending on the tissue and cellular context. In addition, this pathway is one of the most activated in cancer cells and contributes to metastasis (Venkatesh et al., 2018). For example, the development of squamous cell carcinomas in various epithelial tissues is directly related to mutations in members of the Notch family. These mutations represent the most common cause of misregulation of this signaling pathway (Nowell and Radtke, 2017).

The Wnt signaling pathway regulates the embryogenesis and homeostasis of multicellular organisms. In sea urchin embryos, the Wnt signaling pathway contributes to the activation of the endomesodermal gene regulatory network, whose genes start their expression on the 16-cell stage of embryos (Kumburegama and Wikramanayake, 2008). Also, this pathway regulates the formation of the animal–vegetal (A–V) axis in sea urchin and sea anemone embryos.

In humans, cancer, obesity, and diabetes are the result of the Wnt pathway dysregulation (Langton et al., 2016; Mzoughi et al., 2017). It is shown that the pathway is involved in the regulation of the metabolism of cancer cells, which facilitates tumor progression (Lu et al., 2010; Wang and Kunze, 2015; Poli et al., 2018; Sun et al., 2018).

In the comparison analysis, it was shown that the sea urchin genome contains about 90% of the described homologous components of Wnt signal transduction pathways (Croce et al., 2006; Robert et al., 2014). However, from 13 known Wnt subfamilies, *S. purpuratus* has only 11: it is missing only Wnt2 and Wnt11 homologs. Meanwhile, last year Croces’ group identified a gene encoding Wnt2 ortholog in the genome of a related sea urchin *P. lividus*. However, they found no evidence of a bona fide wnt2 gene in *S. purpuratus* when they reanalyzed its genome (Robert et al., 2019). A consortium of scientists was able to find only about half of the Wnt transcriptional target genes that were reported in the literature.

The purple sea urchin can also be considered as a model of gene expression in the normal developmental processes and is used now as an *in vivo* model to evaluate the Epithelial/Mesenchymal Transition (EMT; Romancino et al., 2017). In humans, reactivated EMT drives organ fibrosis and tumor progression (Lim and Thiery, 2012; Wu et al., 2012; Nieto et al., 2016). The process of EMT is regulated by a cohort of specific transcription factors that includes Zeb1/Zeb2, Snail, Slug, and Twist (Tulchinsky et al., 2019). Together with chromatin-modifying enzymes, these factors exert both repressive and activating functions. For example, Zeb1, by binding to the E-box consensus site in the DNA, inhibits the transcription of the CDH1 gene, whose product plays a critical role in forming cell-cell junctions. On the other hand, when Zeb1 complexes with Yap1, a member of the Hyppo pathway, it becomes a transcriptional activator to control the transcription of CTGF and AXL genes (Lehmann et al., 2016).

## The Postgenomic ERA in Sea Urchin-Related Research

### Sea Urchin Genome Sequencing and Analysis

As mentioned before, the sea urchin genome reveals striking similarities to humans and shares with the latter a lot of common gene regulatory pathways.

The first assembly and annotation of the sea urchin genome results were published in 2006 (Sea Urchin Genome Sequencing Consortium et al., 2006) and initiated an active exploration of its genomics and transcriptomics. Over the past 14 years, researchers refined the assembly and annotation of the sea urchin genome. Additional genomic and transcriptomic resources were created, for example, EchinoBase.^1^

The primary assessment of the purple sea urchin genome was estimated as ∼800 Mb in size (Hinegardner, 1971). After the deep-sequencing refinement, the purple sea urchin genome was predicted to contain 23,300 genes (Sea Urchin Genome Sequencing Consortium et al., 2006). The current genome analysis revealed 33,491 genes (and 556 pseudogenes) that encode 38,439 proteins.^2^

A comparative analysis of the sea urchin genome with vertebrates revealed an unprecedented complexity relative to other animals in terms of their innate immune recognition receptors (Rast et al., 2006). The SUGSC research team assumed that about 4–5% of all the sea urchin genes identified are involved directly in the immune functions (Sea Urchin Genome Sequencing Consortium et al., 2006).

Around 222 members of the Toll-like receptor family and 203 genes of the NACHT domain–LRR family were described in the sea urchin genome in addition to genes from a large family of cysteine-rich receptor proteins (Rast et al., 2006). The sea urchin immune system showed the presence of a complement system similar to the chordate (Hibino et al., 2006).

Since transcriptional networks are regulated by transcription factors, it is important to mention the work of Howard-Ashby’s team that described the main families of genes coding for transcription factors in the *S. purpuratus* genome (bHLH, Nuclear Receptor, Basic Leucine Zipper, T-box, Smad, Sox, and other smaller families). The number of genes encoding transcription factors of each family in the sea urchin is comparable to that found in the Drosophila genome, but it is almost twice less than the number of such genes found in the human genome. A similar result was obtained when analyzing the situation with HomeoBox genes (Howard-Ashby et al., 2006). The evolvement of new genes during the evolution since branching Echinodermata from the common Deuterostome branch is associated with the adaptation process, increasing the level of complexity and/or changing the key cellular mechanisms.

During the course of genome analysis, the SUGSC team identified more than 1,200 genes involved in signal transduction. The *S. purpuratus* genome contains 353 protein kinases (Sea Urchin Genome Sequencing Consortium et al., 2006) and 14 lipid kinases (Bradham et al., 2006). The number of sea urchin protein kinases is higher than that described in the Drosophila genome (about 230 members) but less than in the human kinome (518). Although most of the sea urchin kinase subfamilies are often represented by only a single member, their diversity is surprisingly high and corresponds to approximately 97% of the whole human kinome. Only four subfamilies of kinases are missing (Axl, FastK, H11, and NKF3) in the sea urchin compared to the human kinome, whereas the fruit fly kinome lacks 20 of those, and the kinome of worms misses 32 subfamilies (Bradham et al., 2006). Importantly, it has been shown that approximately 88% of described kinases are expressed during embryogenesis (Bradham et al., 2006; Byrum et al., 2006).

To follow the compilation of similarities in the gene ontology of the sea urchin and of humans, it is important to note that they share common mechanisms of cell cycle control. Perhaps, not surprisingly, a number of genes involved in cell cycle control and DNA metabolism have been described for the sea urchin, although its number is lower compared to the human genome. In addition, a few cases of echinoderm-specific gene diversifications have been described (Fernandez-Guerra et al., 2006). Notably, the sea urchin genome contains orthologs of almost all cyclin-dependent kinases, except CDK3. Members of the NIMA-related kinases family (NEK proteins) are, judging by their complexity, close to vertebrates, whereas the complexity of Polo and Aurora mitotic kinase families are close to those found in the worm (Fernandez-Guerra et al., 2006).

Furthermore, a number of known genes involved in DNA replication, repair, and the mitotic checkpoint were also found in the sea urchin. Interestingly, the sea urchin has a single p63/p73 hybrid homologous to the p53, p63, and p73 members of the p53 family of tumor suppressors (Belyi et al., 2010). In addition, the sea urchin contains two homologs of the pRB tumor suppressor and also one homolog of the p21/p27 family of CDK inhibitors (Fernandez-Guerra et al., 2006). Furthermore, the sea urchin genome shares four families of RAS GTPases with humans: Ras, Rho, Rab, and Afr, although 90% of all small GTPases are expressed during embryogenesis (Beane et al., 2006).

### Gene Regulatory Networks

As early as in the pre-genome era, common features, and concepts, of the gene regulatory network (GRN) were described by researchers who used the *S. purpuratus* sea urchin as a model organism. Genome sequencing and annotation made it possible to structure the information, which led to the creation of one of the most complete networks for the regulation of genes during the early embryogenesis (Davidson et al., 2002; Sea Urchin Genome Sequencing Consortium et al., 2006; Oliveri et al., 2008; Peter and Davidson, 2010, 2017; Peter et al., 2012; Martik et al., 2016).

The key step toward the understanding of basic mechanisms of embryogenesis and global GRN was the deep sequencing of RNA. This allowed the accumulation of data on gene expression networking during the embryogenesis of *S. purpuratus* (Rafiq et al., 2014; Tu et al., 2014; Barsi et al., 2015; Gildor et al., 2016; Israel et al., 2016; Janies et al., 2016; Pérez-Portela et al., 2016). The described schemes of the GRN became the best tool for the analysis of the development of the genetic control (Peter and Davidson, 2017).

In the study by Tu et al. (2014), the expression profiles of more than 16,000 genes were measured during embryogenesis. For a clearer presentation of the expression profiles of embryonic genes, the authors performed a cluster analysis. The clusters were grouped into four main groups according to their overall dynamics: “off,” “on,” “transient,” and “other” (Tu et al., 2014). They showed that complex expression patterns of many genes underlie embryonic development, especially in the early stages preceding gastrulation.

A study of Rafiq (2014) sets a basis for understanding the genomic regulatory control of a major morphogenetic process – skeletal morphogenesis for embryogenesis. The authors have identified 420 transcripts whose expression levels in primary mesenchymal cells (PMC) were significantly different from other samples. Most of these genes are transcribed at relatively low levels at the stage of mesenchymal blastula. They were targeted at Ets1 and Alx1, key transcription factors that provide regulatory inputs at the top of the PMC regulatory differentiation network.

It was shown that more than half of the identified transcripts received essential inputs from Ets1 and/or Alx1, most of which were positive. All these data point to their key role in the cell-specific identity of PMCs (Rafiq et al., 2014). Additionally, the authors described about 200 transcripts that were not significantly affected by Ets1 or Alx1 knockdown.

The name of the GRN concept implies that exons play a major role in gene cascades. However, The Human Genome Project and the subsequent deciphering of a large number of genomes made it obvious that the bulk of the genome consists of sets of repetitive DNA (Lander et al., 2001). Coding DNA fragments (exons) occupy no more than 2–3% of the genome (Carey, 2017). The major components of the genome are represented by two groups of repetitive DNA sequences: tandem repeats and dispersed repeats or transposons [transposable elements, (TE); Paço et al., 2019]. Almost all eukaryotic genomes contain TE, for example, it occupies about half of the human genome (Wang and Kunze, 2015).

### Non-coding RNA in the Sea Urchin

It is well established that almost all of the human genome is transcribed into RNA. Surprisingly, most of the transcribed RNA does not code for proteins and is called non-coding RNA (ncRNA; Lander et al., 2001; Carninci et al., 2005; ENCODE Project Consortium et al., 2007; Djebali et al., 2012; Laurent et al., 2015; Carey, 2017), including microRNA (miRNA, 22–25 bp) and long non-coding RNA (lncRNA, <200 bp). NcRNAs are involved in the transcription regulation of genes and other ncRNA (Li and Liu, 2019). The primary source of all kinds of ncRNA in the genome is transposons (Hadjiargyrou and Delihas, 2013).

MicroRNAs control gene expression *via* multiple modes (Cai et al., 2009; Steitz and Vasudevan, 2009). In general, the 5' proximal “seed” region (nucleotide 2–8) of miRNAs exhibits imperfect complementarity to the 3’UTR of the target mRNA (Lewis et al., 2005). Subsequently, this newly formed double-stranded RNA is destroyed by dsRNAse, RISC. However, a few cases have been reported when miRNAs regulated the expression by binding the 5'UTR of mRNAs, thereby interfering with the binding of translation initiation factor, eIF4 (Lee et al., 2009; Brümmer and Hausser, 2014).

To date, several models have been proposed describing the consequences of the interaction between the miRNA complex and their targets. The miRNA-dependent gene silencing can be achieved at three stages, including pre-translational, co-translational, and post-translational steps (Finnegan and Matzke, 2003; Garneau et al., 2007; Grewal and Elgin, 2007; Fabian et al., 2010; Carroll et al., 2014).

The lncRNA-driven transcriptional regulation is more complex and includes several mechanisms: (1) lncRNA can recruit a regulatory protein complex to a gene or an entire chromosome; (2) the binding of a transcriptional factor is inhibited by lncRNA; (3) transcription of lncRNAs regulates the transcription of adjacent protein-coding genes; and (4) the heterochromatic or euchromatic organization of regions in close proximity stabilizes these territories and controls the spreading of post-translational modifications to nearby chromatin (Long et al., 2017). Furthermore, lncRNAs play a key role in stem cell differentiation, immune response, epigenetic regulation, inflammation-related diseases, and tumor development (Briggs et al., 2015; Huarte, 2015; Boon et al., 2016; Chen et al., 2017).

Description and analysis of ncRNA regulatory networks could provide new insights into gene transcription regulation not only during the embryonic development, but also in cancer, when specific developmental programs are aberrantly reactivated. Thus, understanding the complexity of these regulatory networks will make it possible to determine the consequences of their disruption in the course of various diseases.

## Regulatory Networks Based on Non-Coding RNAs

After the publication of the sea urchin genome annotation, miRNAs were identified (Peterson et al., 2009; Wheeler et al., 2009; Campo-Paysaa et al., 2011). These studies revealed that a few, very conserved, miRNAs are also present in humans (Song et al., 2012). The authors had cloned and sequenced small RNAs (18–40 nucleotides) from different embryo stages, ranging from unfertilized eggs to larva pluteus. Around 49 miRNAs were identified, in which three of these were novel miRNAs (not annotated in miRBase previously). Most of the miRNAs are present in the egg and have dynamic accumulation profiles, with the majority of these being upregulated during gastrulation.

To test the function of miRNA during the embryonic development, authors decided to suppress the dsRNA processing enzyme, Dicer, with Dicer morpholino antisense oligonucleotides (MASO). The majority of injected embryos successfully developed to the stage of blastula. However, at the stage of 48 h p.f., embryos that expressed Dicer MASO failed to enter the gastrulation stage (Song et al., 2012). Developmental defects varied from general retardation to cell death.

One interesting example of opposing functions exerted by one micrRNA is miR-31. Importantly, it is expressed during the embryogenesis of *S. purpuratus* and suppresses several components of PMC GRN (Pmar1, Alx1, Snail, and VegfR7). Knockdown of miR-31 causes a disturbance of the function of PMC that forms the embryonic skeleton (Stepicheva and Song, 2015). Meanwhile, in humans, miR-31 is considered as a tumor suppressor. Yet, it can affect several signaling pathways that have opposite effects on the proliferation: RAS/MARK and PI3K/AKT stimulate growth, whereas RB/E2F inhibits it. Unfortunately, specific molecular mechanisms that regulate miR-31 in the sea urchin are not known at the moment.

**Figure 4:**
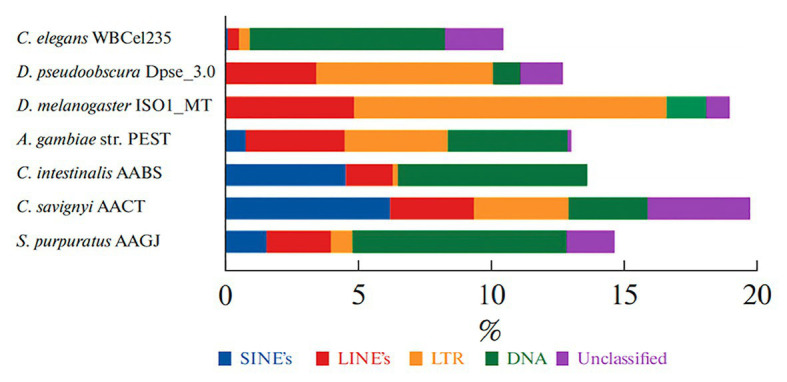
The percent content of transposable elements (TE) in genomes of the sea urchin *S. purpuratus* and other invertebrates. TE classes are marked with a color: nonLTR SINE – blue; LINE – red; LTR TE – yellow; DNA transposons – green; non-annotated repeats – lilac (from Lebedev et al., 2019).

The latest analysis of the sea urchin genome showed that known transposons^3^ occupy about 15% of the genome, including the major class DNA transposons (Figure 4; Lebedev et al., 2019). The percentage of genome occupied by transposons is higher than that of the worm *Caenorhabditis elegans* but less than that of fruit fly or human (Kazazian and Moran, 2017).

The development of new sequencing techniques, such as RNA-Seq, has greatly advanced our understanding and knowledge of new RNAs. In one of the latest studies on this, Hezroni and co-authors, using a PLAR-algorithm, predicted more than 5,000 new sequences of lncRNA in sea urchin transcriptomes (Hezroni et al., 2015).

Genes that code for lincRNAs are more species-specific and less conserved than the gene encoding proteins. In the genome of the sea urchin, synthenic (homologous genes situated on the same chromosomes but in different species) lncRNA genes were identified for more than 2,000 human lincRNA genes. This suggests that the sea urchin likely contains a lot of conserved functional vertebrate lincRNA homologs (Hezroni et al., 2015). Of all detected syntenic lincRNAs, only 18 were found in other amniotes.

One example is LINC00261, located downstream of the Foxa2 gene, which codes for a transcription factor. In all vertebrates, this lincRNA is expressed in endodermal tissues, and in sea urchins, it is expressed in the gut. LINC00261 plays the tumor suppressive role being involved in the regulation of DNA damage (Shahabi et al., 2019).

Another syntenic lincRNA (partially annotated as LINC01122 and LOC101927285 in humans) is expressed in the brain and reproductive tissues across vertebrates. In sea urchins, it is expressed in the adult ovary. Unfortunately, the specific functions of both orthologs are unknown. However, this region is located between the Fancl and Bcl11a loci and one can assume that ncRNA expressed from this locus, at least in humans, may participate in the regulation of DNA damage response and/or apoptosis possibly through the p53 regulatory network.

Well-known embryogenesis signaling pathways conserved between different species can serve as the starting point for understanding the complex network of development organization. In recent years, ncRNAs have rapidly emerged as crucial regulators of main signaling pathways in embryo development and cancer (Fu et al., 2019). Regulation takes place at different levels: from the transcriptional to the post-transcriptional and translational levels, for example, LncRNA is involved in the regulation of WNT, Notch, and other signaling pathways in cancer (Li and Kang, 2014; Trimarchi et al., 2014; Yuan et al., 2014; Ong et al., 2017; Peng et al., 2017; Shen et al., 2017).

## Future of This Model Organism for Gene Expression Studies

Embryogenesis is regulated by complicated mechanisms to ensure that different types of cells and tissues develop from one cell. All processes of embryogenesis are strictly coordinated by signaling pathways. Next-generation sequencing and bioinformatics methods have made it possible to describe the main components of these pathways (Figure 3) and the branched gene regulatory networks.

Studying the regulatory networks of development and its organization on all levels requires experimental models. It seems to us that this model object – the purple sea urchin *S. purpuratus* – is the most suitable system for studying the regulation system based on ncRNAs. Further deep bioinformatic analysis of the genome, and the transcriptomic profiling of embryogenesis stages of this model object, will promote important discoveries in gene networking.

Spontaneous alterations in these coordinated gene expression programs can lead to the development of an unhealthy embryo. Furthermore, reactivation of these pathways in somatic cells can cause many diseases, including cancer in humans (Table 1).

Interestingly, the history of modern cancer research begins with the sea urchin: in the first decade of the 20th century, the German biologist Boveri discovered that unproper fertilization of sea-urchin eggs with two sperm rather than one led to chromosomal aberrations and to the failure of proper development (Laubichler and Davidson, 2008). Furthermore, purple sea urchins and some other urchin species (*L. variegatus*, *M. franciscanus*) retain the ability to regenerate lost or damaged tissues with age (Bodnar and Coffman, 2016).

However, cancers have not been detected in sea urchins. In fact, the life span of some sea urchin species reaches 100 years (Ebert, 2010; Kober and Bernardi, 2013). How such genomic stability is achieved and what the regulatory transcription mechanisms involved in the longevity of these organisms are require further investigation. Thus, sea urchins can provide insights into the processes in cases of serious human diseases associated with the regulation of transcription.

## Author Contributions

LA drafted the manuscript. AD and NB supervised the preparation of the draft. NB helped with writing, proofreading, and editing the final version of the manuscript. All authors contributed to the article and approved the submitted version.

### Conflict of Interest

The authors declare that the research was conducted in the absence of any commercial or financial relationships that could be construed as a potential conflict of interest.

## Footnotes

^1^http://legacy.echinobase.org/Echinobase/

^2^https://www.ncbi.nlm.nih.gov/genome/86

^3^https://www.girinst.org/repbase
